# P-628. Trends in Influenza- and Pneumonia-Related Mortality in Adults with Septicemia ≥ 25 Years of Age in the United States: A Retrospective Analysis from the CDC WONDER Database, 1999-2020

**DOI:** 10.1093/ofid/ofaf695.841

**Published:** 2026-01-11

**Authors:** Hamza Asif, Saadia Ashraf, Kenneth Hannan

**Affiliations:** University of Louisville Hospital, Louisville, KY; Khyber Teaching Hospital, Peshawar, Pakistan, Peshawar, North-West Frontier, Pakistan; University of Louisville Hospital, Louisville, KY

## Abstract

**Background:**

Sepsis due to influenza and pneumonia is a significant cause of morbidity and mortality. Despite their impact, long-term trends in influenza- and pneumonia-related mortality with co-existing septicemia in the United States (U.S.) have not been thoroughly examined. This study analyzes temporal trends and geographical variations in influenza- and pneumonia-related mortality in the setting of sepsis in adults ≥ 25 years of age from 1999 to 2020.

**Figure:**
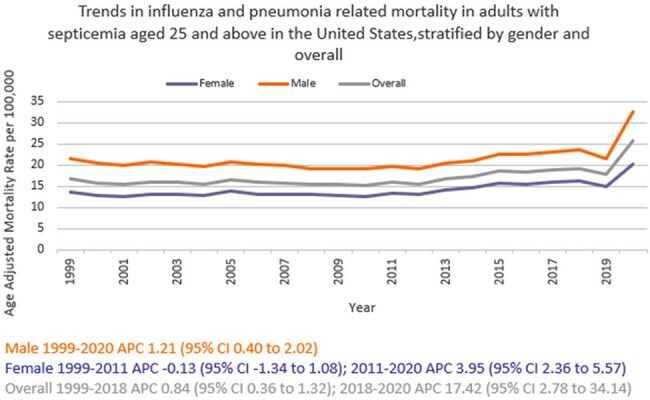

**Figure:**
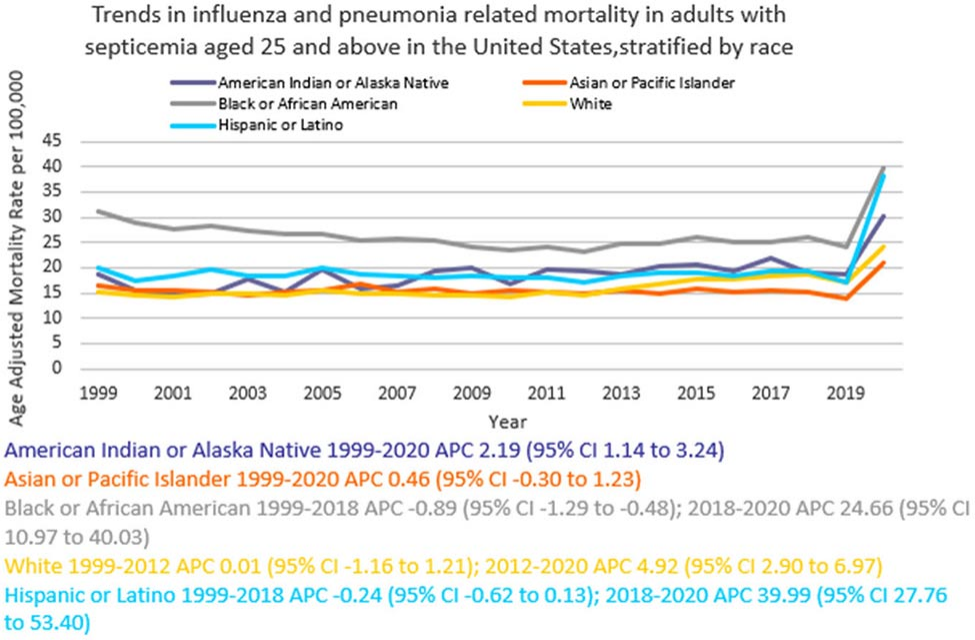

**Methods:**

We analyzed death certificate data from 1999 to 2020 using the the CDC WONDER (Centers for Disease Control and Prevention Wide-Ranging Online Data for Epidemiologic Research) database. Influenza- and pneumonia-related deaths in the context of co-existing sepsis in adults ≥ 25 years of age were examined using ICD 10 codes, with age standardization based on the 2000 U.S. standard population. Mortality rates were expressed as age-adjusted mortality rates (AAMR) per 100,000 population. Joinpoint regression was used to assess trends and calculate annual percentage change (APC), stratified by year, sex, race/ethnicity, census region, type of geographical location, and states.

**Figure:**
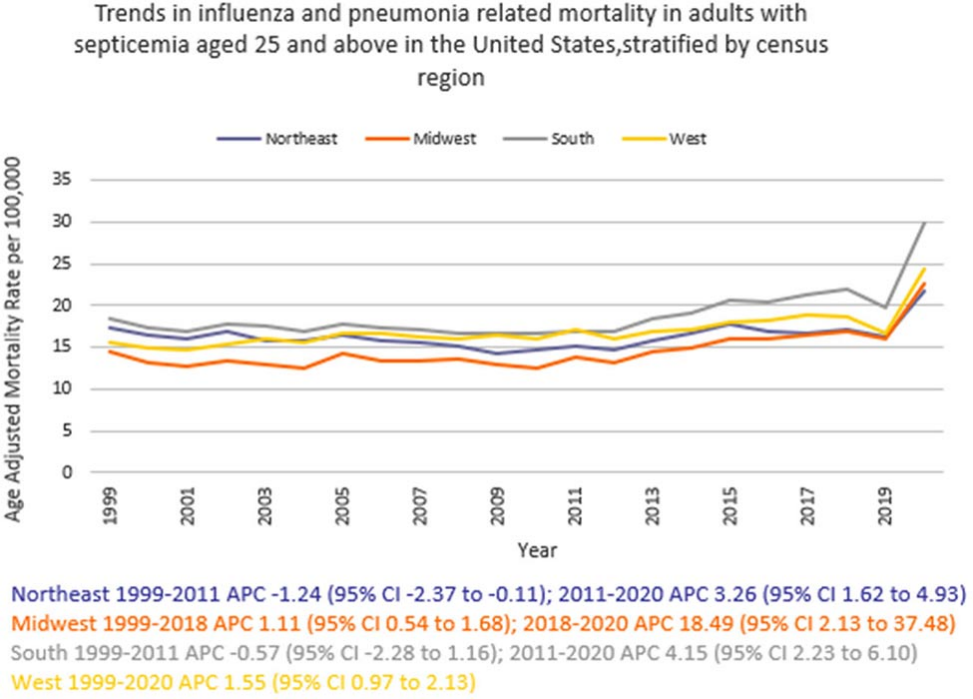

**Figure:**
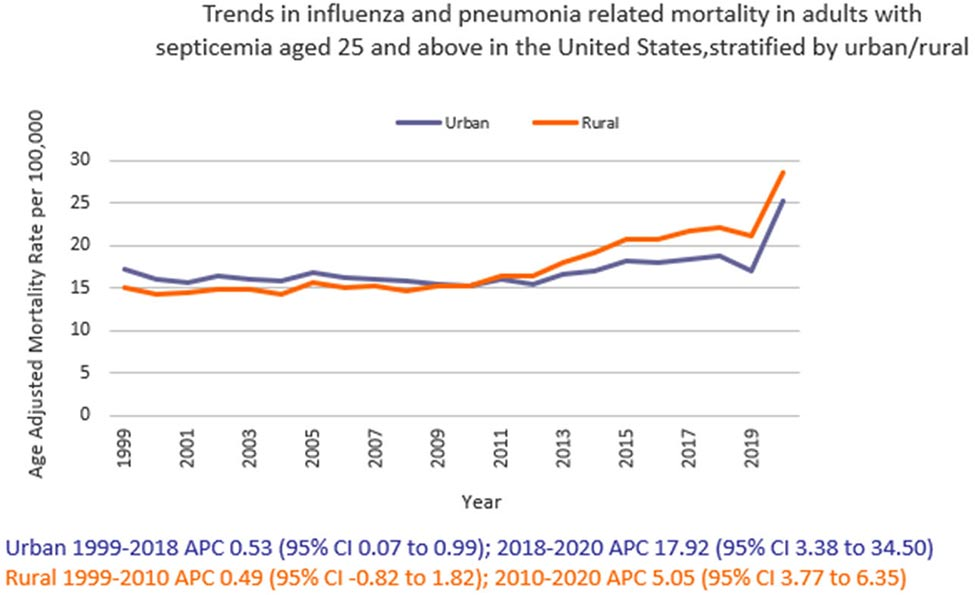

**Results:**

A total of 817,660 related deaths occurred between 1999 and 2020 in the U.S. The AAMR gradually increased from 16.7 in 1999 to 19.2 in 2018 (APC 0.84; 95% CI: 0.36 to 1.32) followed by a sharp increase to 25.6 by 2020 (APC 17.42; 95% CI: 2.78 to 34.14). Men consistently had a higher AAMR than women (21.4 vs. 14.2). Non-Hispanic (NH) Black or African American individuals had the highest AAMR (26.5), followed by Hispanic or Latino (19.9), NH American Indian or Alaska Natives (19.3), NH Whites (16.2), and NH Asian or Pacific Islander (15.6). AAMR was highest in the South (19.1), and in rural areas (17.6) compared to urban areas (17.1). Geographically, AAMRs ranged from 27.1 in District of Columbia to 8.6 in Vermont.

**Conclusion:**

From 1999 to 2020, mortality from influenza and pneumonia with septicemia in the U.S. trended upwards overall, with persistent disparities disproportionately affecting men, non-Hispanic Blacks, and those living in the South and in rural areas. These findings highlight the need to addressing these disparities through targeted interventions in order to decrease the overall burden of mortality, especially amongst vulnerable groups.

**Disclosures:**

All Authors: No reported disclosures

